# Role of lone-pair electron localization in temperature-induced phase transitions in mimetite

**DOI:** 10.1107/S2052520622006254

**Published:** 2022-07-09

**Authors:** Georgia Cametti, Mariko Nagashima, Sergey V. Churakov

**Affiliations:** a Institute of Geological Sciences, University of Bern, Baltzerstrasse 1+3, Bern, 3012, Switzerland; b Graduate School of Sciences and Technology for Innovation, Yamaguchi University, 753-8512, Yamaguchi, Japan; c Paul Scherrer Institut, Forschungstrasse 111, Villigen, 5232, Switzerland; Siberian Branch of Russian Academy of Science, Russian Federation

**Keywords:** mimetite, ELF, phase transition, apatite structure-type, Wannier centres

## Abstract

The crystal structure of mimetite Pb_5_(AsO_4_)_3_Cl, a phosphate with apatite structure-type has been investigated *in situ* at 123, 173, 273, 288, 353 and 393 K. A careful inspection of the diffraction pattern and subsequent structure refinements indicated that mimetite transforms from the monoclinic to the hexagonal polymorph with increasing temperature. At 123 K, a monoclinic superstructure, mimetite-2M, with cell parameters *a* = 20.4487 (9),  *b* = 7.4362 (2), *c* = 20.4513 (9) Å, β = 119.953 (6)°, *V* = 2694.5 (2) Å^3^ and space group *P*2_1_ was observed. From 173 to 353 K, the reflections of the supercell were evident only along one direction of the corresponding hexagonal apatite-cell and the structure transforms to the polymorph mimetite-M with space group *P*2_1_/*b* and unit-cell parameters *a* = 10.2378 (3), *b* = 20.4573 (7), *c* = 7.4457 (2) Å, β = 120.039 (5)°, *V* = 1349.96 (9) Å^3^. Only at higher temperature, *i.e.* 393 K, does mimetite adopt the hexagonal space group *P*6_3_/*m* characteristic of apatite structure-types. The role of the electron lone pairs of Pb atoms in the phase transition was investigated through the analysis of the electron localization function (ELF) calculated based on the DFT-geometry optimized structures of the three polymorphs. The changes in spatial distribution of the 6*s* ^2^ electron density during the phase transitions were explored by means of the Wannier Function Centres (WFCs) derived from *ab initio* molecular dynamics trajectories. In the high-temperature hexagonal structure the 6*s* ^2^ electrons are spherically symmetric relative to the position of Pb atoms. At low temperature the maximum of 6*s* ^2^ electron density is displaced relative to the position of Pb atom contributing to the polar interaction in the monoclinic polymorphs.

## Abstract 2

The monoclinic-to-hexagonal phase transition in mimetite Pb_5_(AsO_4_)_3_Cl was investigated by *in situ* temperature-dependent single crystal X-ray diffraction combined with *ab initio* molecular dynamics simulations.

## 1. Introduction

Minerals of apatite group are the most abundant rock-forming phosphates and the main host of phospho­rus in crustal rocks. Besides, materials with apatite structure-type are used in water purification, bone replacement, ceramic membranes, catalysis, photoluminescence, biomedical applications and many others (Kohn *et al.*, 2002 ▸). The chemical formula of apatite group minerals can be generalized as *A*_5_(*T*O_4_)_3_ *X*, where *A* = large cations, *T* = metals or metalloids, and *X* = anions. The structure can be thought in terms of a zeolite-like topology where one-dimensional tunnels are created by the framework *A*_2_(*T*O_4_)_3_ polyhedra and *A*_3_ *X* represents the extra-framework component. The majority of apatite structural-types crystallize in the hexagonal space group *P*6_3_/*m* (No. 176), where two *A* sites are distinguished: *A*1, which forms the framework network *A*_2_(*T*O_4_)_3_, and *A*2 that is part of the extraframework unit *A*_3_ *X*. Apatite *sensu stricto* refers to three distinct minerals fluorapatite Ca_5_(PO_4_)_3_F, chlorapatite Ca_5_(PO_4_)_3_Cl and hy­droxy­lapatite Ca_5_(PO_4_)_3_OH, described in the space group *P*6_3_/*m* (Hughes *et al.*, 1989 ▸; Hughes & Rakovan, 2002 ▸). Chemical substitutions on cation and/or anion sites can originate several structures, which mostly show sub-symmetries of the hexagonal one.

The presence of Pb^2+^ in the *A* site is of interest for both catalytic and environmental applications. Among them, Pb apatites are used as metal scavengers in treatment of water and contaminated soils, and for the immobilization of radioactive iodine (Wang, 2015 ▸; Cao *et al.*, 2017 ▸). The stereochemical activity of the 6*s* ^2^ lone-pair electrons of Pb plays a crucial role in the structural and electronic properties of Pb apatites (Matar & Galy, 2015 ▸). It is generally accepted that the lone pair cations prefer the *A*2 site in order to reach a more stereochemically flexible environment (Peet *et al.*, 2017 ▸).

Four minerals constitute the isotypic series of Pb apatites: vanadinite, mimetite, pyromorphite, and finnemanite. They all crystallize in the hexagonal space group *P*6_3_/*m* characteristic of apatite (Gabrielson, 1955 ▸; Dai & Hughes, 1989 ▸; Dai *et al.*, 1991 ▸). Interestingly, mimetite Pb_5_(AsO_4_)_3_Cl was also reported to adopt a monoclinic structure, where the *a*_hex_ and *b*_hex_ axes are doubled and the monoclinic angle is close to 120°. The monoclinic polymorph has been solved in different space groups. At first, the polymorph mimetite-M, was described in space group *P*112_1_/*b* (non-standard setting of space group *P*2_1_/*c*, No. 14). The cell parameters imply the doubling of one axis of the hexagonal cell, resulting in: *a* = 10.189 (3), *b* = 20.372 (8), *c* = 7.46 (1) Å, γ = 119.88 (3)° (Dai *et al.*, 1991 ▸). Subsequently, Yang *et al.* (2013 ▸) reported a monoclinic mimetite, mimetite-2M, with a superstructure characterized by the doubling of both *a*- and *b*-axes with respect to hexagonal mimetite (mimetite-H). The symmetry further reduced and the structure was refined in space group *P*2_1_ (No. 4). Similarly, synchrotron single-crystal X-ray diffraction data collected on a natural mimetite, were indexed with a monoclinic superstructure with cell parameters: *a* = 20.44 (1), *b* = 7.437 (1), *c* = 20.44 (1) Å, β = 120° (Baikie *et al.*, 2014 ▸). However, based on the analysis of the systematic absences, the authors opted for the higher-symmetry space group *P*2_1_/*a* (Baikie *et al.*, 2014 ▸). The monoclinic polymorph was observed in both synthetic and natural samples. Studies on mimetite minerals from different localities demonstrated that mimetite-M and -2M are much rarer compared to the hexagonal one. The occurrence of the monoclinic distortion was initially ascribed to changes in temperature: Keppler (1968 ▸, 1969 ▸) reported the phase transition from the monoclinic to the hexagonal polymorphs between 98 and 120°C, depending on the samples. However, these results were not supported by a powder neutron diffraction study of synthetic mimetite that showed no evidence of phase transformation between 293 and 473 K (Baikie *et al.*, 2008 ▸). Later, a transition from monoclinic to hexagonal mimetite was suggested to be both temperature and composition dependent, with minor Ca and P substitutions that would stabilize the hexagonal structure (Baikie *et al.*, 2014 ▸). This conclusion was drawn based on the re-interpretation and re-analysis of previous data (Flis *et al.*, 2010 ▸) on the synthetic solid solution Pb_10_(P_1–*x*_As_*x*_O_4_)_6_Cl_2_. Nevertheless, most mimetite structures (synthetic and natural) were found to be hexagonal at ambient temperature. Thus, it remains unclear what effect the temperature and the chemical composition have on the formation of the monoclinic polymorph.

In this study, we aimed at clarifying different open questions on mimetite. In particular we want to determine: (i) whether the transformation from hexagonal to monoclinic is temperature driven, and (ii) what is the difference between the electronic structure in terms of lone-pair electrons in the two modifications of mimetite and (iii) whether the lone-pairs distribution has an effect on the phase transition. Temperature-dependent X-ray diffraction data were collected *in situ* on a natural sample of mimetite by using a single-crystal X-ray diffractometer to ensure high precision of the structural analysis. To assess the role of chemical bonding and electron localization on the structural transformation in mimetite the maximal localized Wannier centres and electron localization function (ELF) were analysed from atomic trajectories of corresponding polymorphs obtained by *ab initio* molecular dynamic simulations and geometry optimization at DFT level of theory, respectively.

## 2. Methods

### 2.1. Sample description and chemical composition

The sample was a natural mimetite originating from Tsumeb-Otjikoto, Namibia.

The chemical composition was quantitatively determined by electron microprobe analyser (EMPA, Jeol JXA-8230) installed at Yamaguchi University, Japan. The elements, As, P, V, S, Si, Ti, Al, Cr, Fe, Mn, Mg, Ni, Cu, Pb, Ca, Na, K, Sn, F and Cl were measured using an accelerating voltage 15 kV and a beam current of 20 nA with a beam diameter of 1–10 µm. The following probe standards were used: synthetic GaAs (As), synthetic KTiPO_4_ (P), synthetic PbVGe-oxide (V, Pb), natural barite (S), natural wollastonite (Si, Ca), synthetic rutile (Ti), synthetic corundum (Al), synthetic eskolaite (Cr), synthetic hematite (Fe), synthetic manganosite (Mn), synthetic periclase (Mg), synthetic bunsenite (Ni), metallic copper (Cu), natural albite (Na), natural orthoclase (K), natural cassiterite (Sn), synthetic fluorite (F) and synthetic halite (Cl). Wavelength-dispersion spectra were collected using LiF, PET, and TAP monochromator crystals to identify interfering elements and locate the best wavelengths for background measurements. The ZAF correction method was used for all elements. The chemical composition was determined from the average of 31 analytical points and calculated based on 12 oxygen atoms.

### 2.2. Temperature-dependent single-crystal X-ray diffraction

Diffraction data were collected on a Rigaku Synergy-S diffractometer equipped with a double microfocus sources and an Oxford Cryostream-700+ open-flow cryostat. A single fragment of mimetite with dimension 0.100 × 0.06 × 0.04 mm was selected and mounted on the tip of a glass fibre, fixed onto a goniometer head. At first, data were collected at ambient temperature (288 K) by using the Ag *K*α (λ = 0.56087 Å) radiation. Preliminary screening of the crystal indicated a hexagonal unit cell with dimensions *a* = 10.228 (14) Å, *c* = 7.463 (14) Å, *V* = 676 (2) Å^3^. Thus, a data set was collected based on a primitive lattice in the hexagonal Laue class 6/*m*. However, indexing and subsequent data analysis of this data set indicated a monoclinic unit cell with unit-cell parameters: *a* = 10.2354 (13), *b* = 17.7370 (18), *c* = 7.4591 (10) Å, β = 90.099 (13)°, *V* = 1354.2 (3) Å^3^. A new data set was collected based on this monoclinic unit cell, ensuring high redundancy (> 5) and average total *I*/σ(*I*) > 25.

After the data collection at 288 K, the *T*-dependent measurements were performed *in situ* on the same crystal at the following temperatures: on heating at 123, 173, 273, 393 K, and on cooling at 353 K. Between each step, the crystal was equilibrated at the next temperature for at least 30 min. Data collection strategy for each data set was computed based on a monoclinic unit-cell, ensuring high redundancy (*ca* 4) and *I*/σ(*I*) > 20. Data were reduced by using the software package *CrysAlisPro* (Matsumoto *et al.*, 2021 ▸), applying an empirical absorption correction. The structures were solved by direct methods with the software *SHELXS* (Sheldrick, 2008 ▸) and refined by *SHELXL* (Sheldrick, 2015 ▸) using neutral atomic scattering factors.

Data collection and refinement parameters of mimetite at 288 K, 123 and 393 K are summarized in Table 1 ▸. Corresponding experimental parameters of mimetite at 173, 273, and 353 K are reported in Table S1 in supporting information. All structure drawings have been produced by using the software *VESTA* (Momma & Izumi, 2011 ▸).

### 2.3. DFT-based geometry optimization and molecular dynamics simulations

The optimized structures of the three polymorphs (mimetite-H, mimetite-2M, and mimetite-M) were obtained by density functional theory (DFT)-based calculations using the Gaussian and Plane Waves method (GPW) as implemented in the CP2K simulation package (http://www.cp2k.org; Kühne *et al.*, 2020 ▸). The electron exchange and correlations were described by PBE functional (Perdew *et al.*, 1996 ▸). The Kohn–Sham orbitals were expanded using a linear combination of atom-centred Gaussian type orbital functions. In this study, a short-range double-ξ valence polarized basis set for each atom type was used (VandeVondele & Hutter, 2007 ▸). Dispersion correction was taken into account using the DFT+D3 method (Grimme, 2006 ▸).

The initial atomic configuration was taken from the refined structure obtained from XRD experiments. A supercell 2 × 2 × 2 was used for mimetite-H, 2 × 1 × 2 for mimetite-M, and 1 × 1 × 2 for mimetite-2M. The supercell contained 80 Pb, 16 Cl, 192 O and 48 As. The analysis of the lone pair electrons of Pb atoms was performed based on the Electron Localization Function (ELF) (Becke & Edgecombe, 1990 ▸). The ELF values were calculated for the optimized structure of each polymorph based on the definition of Savin *et al.* (1997 ▸), where 0 < ELF < 1 is a normalized function between 0 (zero localization) and 1 (strong localization) with the value ½ corresponding to a free-electron gas behaviour.

To obtain atomic trajectories of the experimentally observed structural polymorphs at different temperatures, a series of MD simulations were performed using the following modelling strategy: The atomic coordinates of mimetite-2M (supercell 2 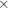 2 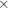 3, to account accurately for dynamic disorder) as obtained by XRD were used as input to run MD simulations at 123 K. After the thermal equilibration for at least 5 ps, atomic trajectories were collected in NPT ensemble at 123 K for 20 ps. The last atomic configuration of the run was used as the input structure to run a second set of NPT MD simulations at 173 K. The same approach was used to subsequently collect the MD trajectories at 293 and 393 K.

For each MD trajectory at 123, 173, 293 and 393 K, the maximally localized Wannier Functions Centres (WFCs) were calculated in step of 100 fs. Structural data analysis was performed by means of *MDAnalysis* software (Michaud-Agrawal *et al.*, 2011 ▸; Gowers *et al.*, 2016 ▸).

## 3. Results and discussion

The chemical composition obtained from EMPA is reported in Table 2 ▸. The analysed crystals were chemically homogeneous and no significant trace of Ca (wt% < 0.01) or P (wt% = 0.15) were detected. This is consistent with the refined chemical formula (Table 1 ▸).

### 3.1. Structural transformations

**Figure 1:**
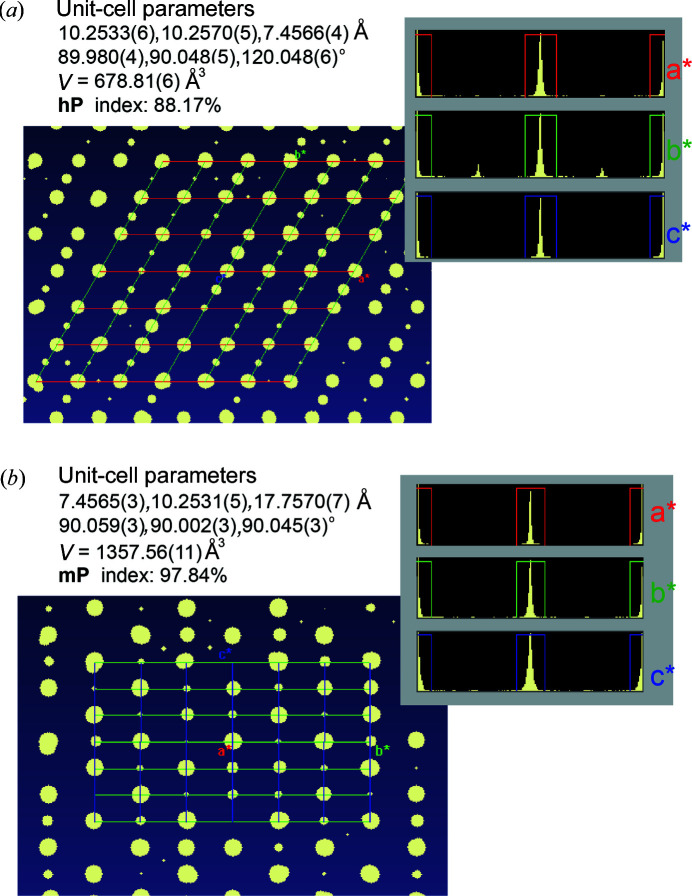
Reciprocal lattice view of mimetite diffraction data collected at 288 K. The distribution histograms of the reflections in the hexagonal (*a*) and monoclinic (*b*) unit cells are shown. The size of the reflection spots is proportional to the intensity of the reflections.

The diffraction pattern of mimetite obtained at ambient temperature and pressure unequivocally indicated the presence of reflections which cannot be indexed by the apatite-type hexagonal unit cell [Fig. 1 ▸(*a*)]. Our analysis pointed to a monoclinic unit-cell, with *c*_m_ = 2*b*_hex_. The doubling of only one unit-cell parameter with respect to the hexagonal cell of mimetite-H corresponds to the mimetite-M polymorph reported by Dai *et al.* (1991 ▸) and reflects the fact that the satellite reflections are mainly located along ½*b*_hex_ axis [Fig. 1 ▸(*a*)]. The monoclinic unit cell, *a* = 10.2552 (3), *b* = 7.45761 (19), *c* = 17.7473 (5) Å, β = 90.038 (3)°, *V* = 1357.30 (7) Å^3^, was used to index the diffraction pattern and to perform the subsequent data reduction [Fig. 1 ▸(*b*)]. Structure solution indicated the space group *P*2_1_/*c* (No. 14), subsequently transformed to the non-standard setting *P*112_1_/*b*, with unit-cell parameters *a* = 10.2552 (3), *b* = 20.4913 (6), *c* = 7.4576 (2) Å, γ = 119.993 (2)°, *V* = 1357.29 (7) Å^3^, for a better comparison with literature data (Dai *et al.*, 1991 ▸). The structure did not show significant differences from mimetite-M previously reported by Dai *et al.* (1991 ▸). Nine oxygen atoms coordinate Pb at the *A*1 sites (Pb1), six are found at distances smaller than 2.8 Å and three at longer distances. Pb atoms at *A*2 sites (Pb2) are on average coordinated by six oxygen and two chlorine atoms. Table 3 ▸ reports the selected bond distances for mimetite-M as calculated form our data and those of mimetite-M taken from Dai *et al.* (1991 ▸).

**Figure 2:**
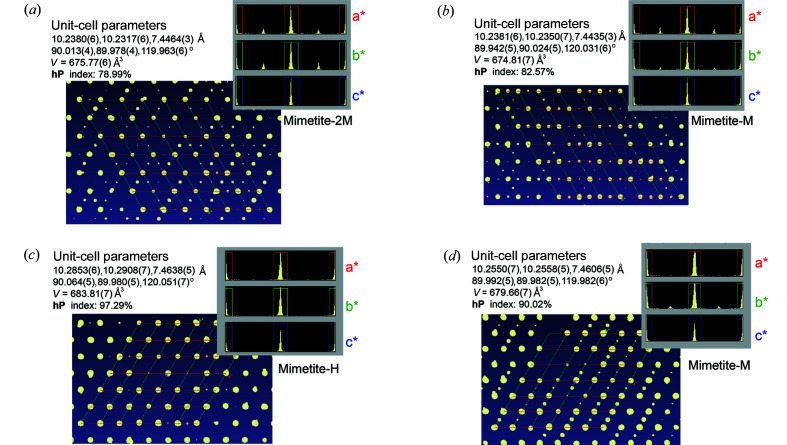
Reciprocal lattice view of mimetite at (*a*) 123, (*b*) 173, (*c*) 393 and (*d*) 353 K. The distribution histograms of the reflections in the hexagonal unit cell are reported at each temperature.

The analysis of the data set measured at 123 K pointed to a monoclinic superstructure with unit-cell parameters: *a* = 20.4487 (9), *b* = 7.4362 (2), *c* = 20.4513 (9) Å, β = 119.953 (6)°, *V* = 2694.5 (2) Å^3^. In this case, the superstructure reflections were clearly distributed along both *a* and *b* axes of the corresponding hexagonal cell [Fig. 2 ▸(*a*)]. The data reduction suggested the monoclinic space group *P*2_1_/*c*. However, due to unsatisfactory structure refinement (negative atomic displacement parameters for oxygen atoms and high residuals close to non-Pb atoms) and the presence of forbidden reflections, an alternative structure refinement was performed in space group *P*2_1_. This structural modification is equivalent to mimetite-2M polymorph described by Yang *et al.* (2013 ▸). Despite the otherwise acceptable agreement criteria, the final refinement was characterized by high difference electron density peaks and an unusually high value for the weighting scheme parameter (Table 1 ▸). This can be explained by the pseudo-symmetry of the structure, *i.e.* the pseudo-centric character as indicated by the refined absolute structure parameter. Baikie *et al.* (2014 ▸) also noticed similar issues in the structure refinement of mimetite at low temperatures. Selected bond distances of mimetite-2M are reported in Table S2. Although small variations in the Pb—O bond distances, the coordination environment of Pb does not change with respect to that of mimetite-M.

At 173 K most of the satellite reflections were evident only along one axis of the corresponding hexagonal-type cell, indicating a gradual change from the 2M to M polymorph [Fig. 2 ▸(*b*)]. Similarly to the data set collected at 288 K, the monoclinic unit-cell *a* = 10.2378 (3), *b* = 7.4457 (2), *c* = 17.7096 (5) Å, β = 89.990 (3)° was refined and subsequently transformed to *a* = 10.2378 (3), *b* = 20.4573 (7), *c* = 7.4457 (2) Å, β = 120.039 (5)°. The structural refinement was performed in space group *P*112_1_/*b* (Table S1). At 273 K, the analysis of the reflections clearly pointed to a monoclinic supercell, with doubling of one hexagonal axis, similar to that found at 173 K and at 288 K. At this temperature, the monoclinic space group *P*112_1_/*b* and the structure of mimetite-M was maintained (Table S1).

After increasing the temperature to 393 K, the structure transformed to the hexagonal polymorph-H, with apatite-structure type (Table 1 ▸). Differently from the data collected at low temperature, no additional reflections, in disagreement with the hexagonal primitive unit cell, were detected in the diffraction pattern [Fig. 2 ▸(*c*)]. It is worth noting that, also in this case the high *I*/σ(*I*) of the collected data ensured the detection of eventual low-intensities reflections belonging to the monoclinic polymorph. When the temperature diminished to 353 K, the satellite reflections became again visible [Fig. 2 ▸(*d*)] and mimetite transforms to the monoclinic structure equivalent to that observed at 173 and 273 K (Table S1).

### 3.2. The role of temperature

Performed analysis indicated that in stoichiometric mimetite, the phase transition from the monoclinic to the hexagonal polymorph is temperature driven. This transition becomes evident with the appearance of satellite reflections, which cannot be indexed by the aristotypic hexagonal unit cell of apatite. The structural changes include two transformations: (i) from mimetite-2M (space group *P*2_1_) to mimetite-M (space group *P*112_1_/*b*) between 123 and 173 K; and (ii) from mimetite-M to mimetite-H (space group *P*6_3_/*m*) between 353 and 393 K. No significant differences were noticed between the mimetite-M polymorphs collected at different temperatures, *i.e.* at 173, 273, 288 and 353 K, as indicated by a unit-cell volume variation smaller than 1%.

In literature, the occurrence of the monoclinic polymorph (both M and 2M) at room temperature was rarely reported. However, structural refinements of mimetite-H at ambient temperature were often associated with high residuals peaks in the difference Fourier maps, large ADPs, and presence of unindexed reflections (Calos *ey al.*, 1990 ▸; Flis *et al.*, 2010 ▸; Baikie *et al.*, 2014 ▸). For instance, in their study on lead-apatite structure types, Baikie *et al.* (2014 ▸) obtained an unsatisfactory structural refinement in *P*6_3_/*m* for a mimetite sample at ambient temperature and brought evidence of a superior fitting performed in the monoclinic space group *P*2_1_/*a*. Based on these observations, it could be suggested that mimetite, at room temperature, always occurs as monoclinic, and the presence of the satellite reflections, due to their lower intensities, was easily overseen. Indeed, data reduction in the hexagonal system (especially if performed rejecting the outlier reflections) and subsequent structural refinement in space group *P*6_3_/*m* may lead to apparently satisfactory results.

Baikie *et al.* (2014 ▸) further demonstrated the effect of the temperature on the monoclinic distortion. The authors reported that the magnitude of the residual peaks in difference Fourier maps and poor refinement indices increased at 100 K with respect the structure measured at ambient temperature.

On the other hand, the influence of the chemical composition must be taken into account as well. The re-analysis of the diffraction data reported by Flis *et al.* (2010 ▸) (Baikie *et al.*, 2014 ▸) demonstrated that the supercell reflections disappear with increasing values of *x* in the Pb_10_(As_1–*x*_P_*x*_O_4_)_6_Cl_2_ solid solution. The effect of the chemical composition is also seen, to a certain extent, in the structural analysis reported by Dai *et al.* (1991 ▸). The authors investigated samples of mimetite from two different localities. The data were collected at ambient temperature by using a comparable exposure time. One sample, with chemical composition (Pb_9.99_)(As_5.74_Si_0.07_S_0.06_P_0.14_)O_24_Cl_2_, was found to show the typical supercell reflections of mimetite-M polymorph with unit-cell parameters *a* = 10.189 (3), *b* = 20.372 (8), *c* = 7.46 (1) Å, γ = 119.88 (3)°. The second sample, identified as hexagonal mimetite-H, had chemical composition (Pb_9.75_Ca_0.32_)(As_5.71_Si_0.13_S_0.13_P_0.01_)O_24_Cl_2_. Thus, small substitutions at Pb sites may also induce the phase transition. Alternatively, it may cause the shift of the transition temperatures toward lower values. The sample used in this study did not contain significant amount of Ca and P impurities, in agreement with the above observations.

### 3.3. The role of the Pb^2+^ 6*s* ^2^ lone pair electrons

The distribution of 6*s* ^2^ electrons of lead atoms in mimetite was investigated by means of the electron localization function calculated from the cell- and geometry-optimized structures of mimetite-M, mimetite-2M and mimetite-H. The unit-cell parameters of the optimized structures, together with the deviation from the experimental XRD data, are reported in Table 4 ▸.

**Figure 3:**
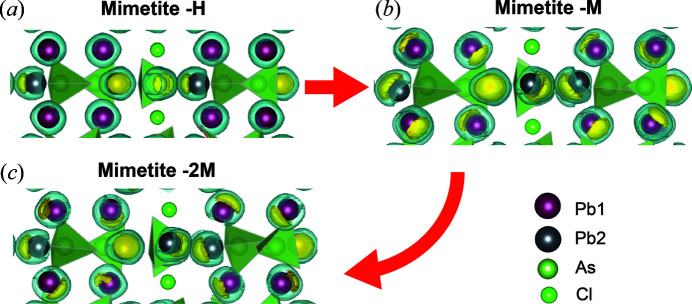
Fragment of the DFT-optimized structures of mimetite-2M, -M and -H (projection along the [110]_hex_, *c*_hex_-axis vertical). ELF isosurfaces are reported in yellow and light blue for ELF = 0.96 and ELF = 0.90, respectively. As tetrahedra are shown in light green, Cl atoms in green. Dark-grey and purple spheres represent Pb atoms residing at Pb2 and Pb1 crystallographic sites, respectively.

Fig. 3 ▸ shows the isosurfaces of the ELF in the three polymorphs. Similar to the data reported by Peet *et al.* (2017 ▸), our calculations indicated that, in mimetite-H, the lone-pair electrons of Pb2 sites [Fig. 3 ▸(*a*)] are more localized than those at Pb1. The localization of the electron pairs at Pb2 remains overall unaltered among the different polymorphs, suggesting that the temperature has no influence on the Pb2 lone pairs. In contrast, differences in the ELF close to Pb1 sites were observed among mimetite-M, -2M and -H.

In mimetite-H, the 6*s* ^2^ electrons of Pb1 site are more delocalized with respect to those in mimetite-M and -2M [Figs. 3 ▸(*b*) and 3 ▸(*c*)]. A general gradual localization of the lone pair electrons at Pb1 is observed from the higher (*i.e.* mimetite-H) to the lower (*i.e.* mimetite-2M) symmetry phase. Mimetite-M is characterized by an increased localization of 6*s* ^2^ at each Pb1 site with respect mimetite-H [Fig. 3 ▸(*b*)]. In mimetite-2M [Fig. 3 ▸(*c*)], 6*s* ^2^ electron pairs further localize at Pb1 sites corresponding to Pb11, Pb14, P15 and Pb17 sites of the refined structure (cif provided in supporting information). Thus, the increased localization does not affect equally all Pb1 sites and this might explain the monoclinic distortion at low temperatures.

**Figure 4:**
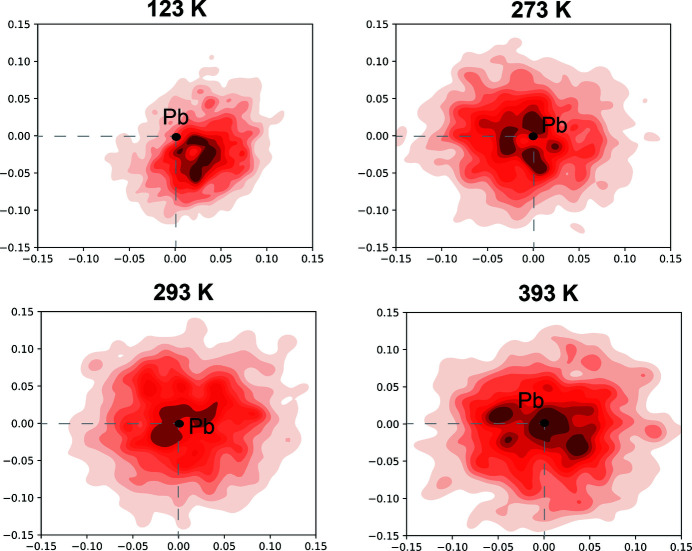
Density plots of the WFCs for selected Pb atoms corresponding to six equivalent Pb1 sites in the supercell of the MD trajectories calculated at 123, 273, 293 and 393 K. The plots show the density distribution (*x* versus *z* coordinates) of the WFCs extracted from MD trajectories in steps of 100 fs. The WFCs coordinates are expressed as a difference with respect the reference Pb coordinates.

This observation was further confirmed by the analysis of the WFCs computed from MD trajectories at different temperatures. To get an insight on the dynamic of the lone-pair electrons, the relative positions of the Wannier centres, representing 6*s* ^2^ states with respect to corresponding Pb core position, have been sampled from MD trajectory with a 100 fs interval for each data set (*i.e.* 123, 273, 293, 393 K). The analysis was focused on the Pb atoms residing at Pb1 crystallographic sites that are suggested to have distinct localization in different polymorphs according to ELF. The *x* versus *z* coordinates (in the simulated cell the *a*,*b* axes of the hexagonal cell correspond to **a**,**c** directions) of the extracted WFCs, at different time steps, for the selected Pb atoms were plotted to visualize the distribution of the lone-pair electrons in the (010) plane. In this way, we can observe the relative distribution of the WFCs centres with respect to reference Pb core and their spread. As an example, in Fig. 4 ▸ the density plot, for representative Pb1 atoms, is reported for the simulated structures at 123, 273, 293 and 393 K. In agreement with the prediction of ELF for the optimized structures, the electron pairs show a spherical and broader distribution around the Pb at higher temperatures. In contrast, the plot of the data set calculated at 123 K shows that the centre of WFCs density distribution is clearly displaced off from the location of Pb core.

**Figure 5:**
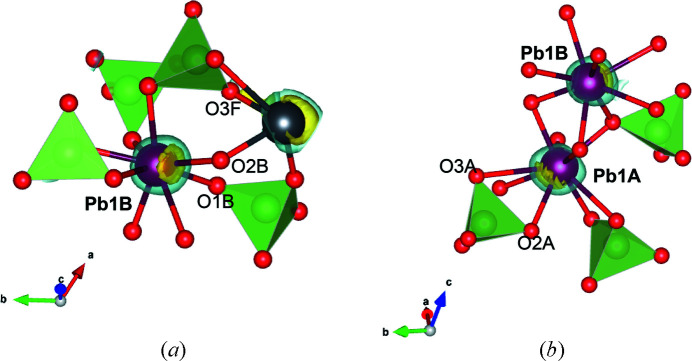
Detail of the DFT-optimized structure of mimetite-M showing the position of the ELF isosurfaces for Pb1*B* (*a*) and Pb1*A* sites (*b*). Colour code as in Fig. 3 ▸.

Dai *et al.* (1991 ▸) already suggested, based on the geometric parameters, that ‘a redistribution of the lone-pair electrons associated with Pb1’ occurred as a result of the phase transformation from the hexagonal to the monoclinic structure. In particular, the authors stated that the lone-pair electrons of Pb^2+^ located at Pb1 sites (Pb1*A* and Pb1*B* symmetry-independent in the monoclinic structure) shifted toward O3*A* (for Pb1*A*) and O3*F* (for Pb1*B*) in mimetite-M. Our analysis partially agreed with this description. The isosurfaces of the ELF at Pb1*B* showed that the 6*s* ^2^ electrons are mostly shifted toward O2*B* instead of O3*F* [Fig. 5 ▸(*a*)], whereas those of Pb1*A* are mostly localized between O3*A* and O2*A* [Fig. 5 ▸(*b*)].

The phase transitions in mimetite can be related to the change of the stereochemical activity of the lone pair electrons of the Pb1 site. The same phenomenon was observed in other Pb-containing crystalline compounds. The perovskite-like structure of Pb_2_MgWO_6_ undergoes an antiferroelectric phase transition below 310 K from cubic to orthorhombic. Such transition is accompanied by a relocation of the lone pairs of lead atoms, ‘which are smeared out by the near –octahedral symmetry of the lead sites in the high-temperature phase whereas in the low temperature phase they are localized into the traditional lobes’ (Seshadri *et al.*, 1999 ▸). The authors associated the phase transition with a tendency of the lone pair electrons to localize close to the average position of Pb core. Similarly, the transitions and related structural changes in BiFeO_3_ and PbTiO_3_ are mainly provoked by the stereochemical activity of the lone-pair two valence *s*-electrons of Bi^3+^ and Pb^2+^ (Volkova & Marinin, 2011*a* ▸,*b* ▸). The same authors demonstrated that the increase in temperature (or pressure) diminishes the stereochemical activity of the lone-pair electrons, resulting in a decreased distortion of the cations coordination surroundings (Volkova & Udovenko, 1988 ▸; Volkova & Marinin, 2011*a* ▸,*b* ▸).

If the localization of the semi-local states of Pb controls the phase transition, this would also explain the absence of the supercell reflections in Ca-rich mimetite samples.

## 4. Conclusions

The *in situ* measurements brought evidence that the transformation in the sample of stoichiometric mimetite from monoclinic to hexagonal and *vice versa* are definitively temperature dependent. The polymorph mimetite-2M is observed only at low temperatures (below 173 K). From 173 to 353 K mimetite transforms to mimetite-M, and only at temperatures above 373 K the supercell reflections disappeared leading to the hexagonal structure, mimetite-H. Based on our results, we speculate that the majority of mimetite samples are monoclinic at ambient temperature. The presence of the additional reflections of the monoclinic supercell can be easily overlooked without a careful inspection of the reciprocal lattice.

The observed structural changes are related to the stereochemical activity of the lone pair electrons of the Pb at Pb1 site. The computed ELF for the three polymorphs unambiguously showed that the 6*s* ^2^ lone pair progressively localizes close to the Pb core as the temperature decreases, influencing the distortion of the coordination geometry of the Pb atoms. As previously reported for other other compounds showing similar stereochemical activity of lone pair, the charge localization acts as a stabilizing factor at lower temperatures.

These results clarify the role of temperature on the crystal symmetry of natural mimetite. Concerning the effect of the chemical compositions (presence of Ca and P impurities), we can draw some conclusions based on data published in literature. It was shown that the higher the deviation from a chemically pure mimetite composition, the less evident are the supercell reflections indicative for the monoclinic space group. Hence, we can hypothesize that the transition from the hexagonal to the monoclinic phases is progressively shifted toward lower temperatures with increasing P and Ca content in mimetite crystals.

## Supplementary Material

Crystal structure: contains datablock(s) Mim123K, Mim173K, Mim273K, Mim288K, Mim353K, Mim393K. DOI: 10.1107/S2052520622006254/tq5003sup1.cif

Experimental details of mimetite at 173, 273 and 353K (Table S1) and selected geometric parameters of mimetite at 123K (Table S2). DOI: 10.1107/S2052520622006254/tq5003sup2.pdf

Structure factors: contains datablock(s) Mim123K. DOI: 10.1107/S2052520622006254/tq5003Mim123Ksup3.hkl

Structure factors: contains datablock(s) Mim173K. DOI: 10.1107/S2052520622006254/tq5003Mim173Ksup4.hkl

Structure factors: contains datablock(s) Mim273K. DOI: 10.1107/S2052520622006254/tq5003Mim273Ksup5.hkl

Structure factors: contains datablock(s) Mim288K. DOI: 10.1107/S2052520622006254/tq5003Mim288Ksup6.hkl

Structure factors: contains datablock(s) Mim353K. DOI: 10.1107/S2052520622006254/tq5003Mim353Ksup7.hkl

CCDC references: 2178919, 2178920, 2178921, 2178922, 2178923, 2178924

## Figures and Tables

**Table 1:** Experimental details for mimetite at 288, 123 and 393 K

	Mimetite-M (288 K)	Mimetite-2M (123 K)	Mimetite-H (393 K)
Crystal data			
Chemical formula	As_2_Cl_0.67_O_8_Pb_3.33_	As_3_ClO_12_Pb_5_	As_3_ClO_12_Pb_5_
Crystal system, space group	Monoclinic, *P*112_1_/*b*	Monoclinic, *P*2_1_	Hexagonal, *P*6_3_/*m*
*a*, *b*, *c* (Å)	10.2552 (3), 20.4913 (6), 7.4576 (2)	20.4487 (9), 7.4362 (2), 20.4513 (9)	10.2722 (3), 7.4537 (3)
β (°)	90	119.953 (6)	90
γ (°)	119.993 (2)	90	120
*V* (Å^3^)	1357.29 (7)	2694.5 (2)	681.14 (5)
*Z*	6	6	2
Radiation type, λ (Å)	Ag *K*α, 0.56087	Ag *K*α, 0.56087	Ag *K*α, 0.56087
μ (mm^−1^)	37.74	37.49	37.60
Crystal size (mm)	0.10 × 0.06 × 0.04	0.10 × 0.06 × 0.04	0.10 × 0.06 × 0.04
		
Data collection			
Diffractometer	XtaLAB Synergy, Dualflex, Hypix	XtalLAB, Synergy, Dualflex, Hypix	XtalLAB, Synergy, Dualflex, Hypix
No. of measured, independent and observed [*I* > 2σ(*I*)] reflections	27930, 5638, 4670	50947, 19430, 15378	11659, 1202, 1060
*R*_int_	0.048	0.056	0.038
(sin θ/λ)_max_ (Å^−1^)	0.794	0.769	0.870
		
Refinement			
*R*[*F* ^2^ > 2σ(*F* ^2^)], *wR*(*F* ^2^), *S*	0.033, 0.078, 1.06	0.055, 0.129, 1.08	0.027, 0.059, 1.14
No. of reflections	5638	19430	1202
No. of parameters	190	387	39
No. of restraints		1	
	*w* = 1/[σ^2^(*F*_o_^2^) + (0.0292*P*)^2^ + 12.8986*P*] where *P* = (*F*_o_^2^ + 2*F*_c_^2^)/3	*w* = 1/[σ^2^(*F*_o_^2^) + 226.143*P*] where *P* = (*F*_o_^2^ + 2*F*_c_^2^)/3	*w* = 1/[σ^2^(*F*_o_^2^) + (0.0248*P*)^2^ + 4.3504*P*] where *P* = (*F*_o_^2^ + 2*F*_c_^2^)/3
Δρ_max_, Δρ_min_ (e Å^−3^)	4.21, −4.54	6.42, −11.99 (see comment in §3.1)	2.35, −3.92
Absolute structure	–	Refined as an inversion twin	–
Absolute structure parameter	–	0.51 (5)	–

**Table 2:** Average composition of mimetite obtained from EMPA apfu is atoms per formula unit.

	wt%	Std. Dev.			apfu	Std. Dev.
As_2_O_5_	22.64	0.24		As^5+^	2.72	0.02
P_2_O_5_	0.15	0.12		P^5+^	0.03	0.02
V_2_O_5_	0.01	0.02		V^5+^	0.00	0.00
SO_3_	0.01	0.02		S^6+^	0.00	0.00
SiO_2_	0.01	0.01		Si	0.00	0.00
TiO_2_	0.02	0.02		Ti	0.00	0.00
Al_2_O_3_	0.01	0.01		Al	0.00	0.00
Cr_2_O_3_	0.01	0.02		Cr^3+^	0.00	0.00
Fe_2_O_3_	0.01	0.02		Fe^3+^	0.00	0.00
MnO	0.00	0.01		Mn^2+^	0.00	0.00
MgO	0.02	0.01		Mg	0.01	0.00
NiO	0.02	0.03		Ni	0.00	0.00
CuO	0.00	0.01		Cu^2+^	0.00	0.00
PbO	73.84	0.62		Pb^2+^	4.58	0.02
CaO	0.01	0.01		Ca	0.00	0.00
Na_2_O	0.01	0.02		Na	0.00	0.01
K_2_O	0.00	0.00		K	0.00	0.00
SnO_2_	0.02	0.02		Sn^4+^	0.00	0.00
F	0.02	0.03		F^−^	0.01	0.02
Cl	2.47	0.08		Cl^−^	0.96	0.03
Total	99.28					
Ex. O†	0.57					
Total − Ex. O	98.71					

† Excluded oxygen content corrected for Cl and F substitution.

**Table 3:** Selected geometric parameters of mimetite-M at 288 K (Å) The second line show the corresponding distances reported by Dai *et al.* (1991 ▸).

Pb1*A*—O1*B* ^i^	2.476 (5)	Pb1*B*—O3*D*	2.706 (6)	Pb2*C*—O2*B* ^i^	2.364 (7)
	2.40 (4)		2.69 (3)		2.38 (3)
Pb1*A*—O1*C*	2.518 (5)	Pb1*B*—O2*B* ^i^	2.829 (8)	Pb2*C*—O3*B* ^vii^	2.443 (7)
	2.54 (3)		2.71 (4)		2.41 (3)
Pb1*A*—O1*A*	2.565 (5)	Pb1*B*—O2*A*	2.853 (6)	Pb2*C*—O3*F*	2.445 (6)
	2.53 (4)		2.81 (3)		2.47 (2)
Pb1*A*—O2*A* ^ii^	2.713 (6)	Pb1*B*—O3*E*	2.952 (9)	Pb2*C*—O1*A* ^viii^	3.077 (6)
	2.73 (2)		3.00 (2)		3.03 (2)
Pb1*A*—O3*C* ^ii^	2.715 (6)	Pb1*B*—O3*F*	3.208 (7)	Pb2*C*—O3*C*	3.078 (7)
	2.68 (3)		3.18 (3)		3.12 (3)
Pb1*A*—O2*B* ^iii^	2.725 (7)	Pb1*B*—Pb1*A* ^i^	3.6289 (3)	Pb2*C*—Cl^viii^	3.1548 (19)
	2.72 (4)				3.14 (2)
Pb1*A*—O3*B* ^ii^	2.929 (10)	Pb1*B*—O1*A* ^i^	2.495 (6)	Pb2*C*—O3*E* ^viii^	2.834 (6)
	2.79 (2)		2.46 (4)		2.85 (2)
Pb1*A*—O2*C* ^ii^	2.931 (6)	Pb1*B*—O1*C* ^i^	2.515 (5)	As*A*—O1*A*	1.672 (6)
	2.94 (2)		2.54 (3)		1.72 (2)
Pb1*A*—O3*A* ^ii^	3.156 (7)	Pb1*B*—O1*B*	2.591 (5)	As*A*—O3*A*	1.683 (6)
	3.12 (3)		2.61 (4)		1.67 (3)
		Pb1*B*—O2*C*	2.659 (6)	As*A*—O3*D*	1.689 (6)
			2.66 (2)		1.69 (3)
				As*A*—O2*A*	1.691 (5)
					1.67 (2)
Pb2*A*—O2*C*	2.362 (5)	Pb2*B*—O2*A* ^i^	2.356 (6)	As*B*—O3*B* ^i^	1.663 (7)
	2.33 (2)		2.33 (1)		1.63 (3)
Pb2*A*—O3*C* ^iv^	2.458 (5)	Pb2*B*—O3*D*	2.497 (6)	As*B*—O3*E* ^i^	1.675 (6)
	2.48 (3)		2.48 (3)		1.67 (2)
Pb2*A*—O3*A*	2.468 (7)	Pb2*B*—O3*E* ^i^	2.596 (9)	As*B*—O1*B* ^ix^	1.677 (5)
	2.51 (2)		2.52 (2)		1.71 (2)
Pb2*A*—O3*F* ^v^	2.805 (6)	Pb2*B*—O3*A* ^vi^	2.795 (6)	As*B*—O2*B*	1.682 (7)
	2.81 (3)		2.79 (3)		1.70 (2)
Pb2*A*—O3*D*	3.016 (8)	Pb2*B*—O3*B* ^i^	2.828 (11)	As*C*—O3*F*	1.682 (5)
	2.97 (3)		3.04 (4)		1.67 (2)
Pb2*A*—O1*B* ^v^	3.077 (7)	Pb2*B*—O1*C* ^i^	3.082 (6)	As*C*—O1*C* ^x^	1.688 (5)
	3.08 (2)		3.08 (2)		1.65 (1)
Pb2*A*—Cl	3.2004 (17)	Pb2*B*—Cl	3.1370 (18)	As_3_—O2*C*	1.691 (6)
	3.19 (2)		3.11 (1)		1.69 (2)
				As_3_—O3*C*	1.692 (5)
					1.63 (3)

Symmetry code(s): (i) −*x* + 1, −*y* + 1, −*z* + 1; (ii) −*x* + 1, −*y* + 1, −*z*; (iii) *x*, *y*, *z* − 1; (iv) −*x*, −*y* + 1, −*z*; (v) −*x*, −*y* + 1, −*z* + 1; (vi) *x*, *y*, *z* + 1; (vii) −*x* + 1, −*y* + 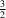 , *z* + 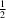 ; (viii) *x*, *y* + 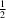 , −*z* + 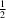 ; (ix) *x*, *y* − 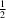 , −*z* + 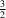 ; (x) *x*−1, *y*, *z*; (xi) *x*, *y* + 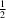 , −*z* + 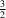 ; (xii) *x* + 1, *y*, *z*; (xiii) −*x* + 1, −*y* + 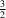 , *z* − 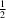 .

**Table 4:** Unit-cell parameters of the DFT-optimized structures of mimetite-2M, -M and -H polymorphs The deviation from the experimental values (single-crystal X-ray diffraction) is reported.

	GEO_OPT	XRD	Deviation (%)
Mimetite-2M			
*a* (Å)	20.764	20.4487 (9)	1.5
*b* (Å)	7.488	7.4362 (2)	0.7
*c* (Å)	20.812	20.4513 (9)	1.8
α (°)	90.0	90	0.0
β (°)	119.975	119.953 (6)	0.02
γ (°)	90.0	90	0.0
*V* (Å^3^)	2802.86	2694.5 (2)	4.0
Mimetite-M			
*a* (Å)	10.419	10.2552 (3)	1.6
*b* (Å)	20.829	20.4913 (6)	1.6
*c* (Å)	7.536	7.4576 (2)	1.0
α (°)	90.0	90	0.0
β (°)	90.0	90	0.0
γ (°)	119.74	119.993 (2)	0.2
*V* (Å^3^)	1420.08	1357.29 (7)	4.6
Mimetite-H			
*a* (Å)	10.388	10.2722 (3)	1.1
*b* (Å)	10.388	10.2722 (3)	1.1
*c* (Å)	7.4434	7.4537 (3)	0.1
α (°)	90.0	90	0.0
β (°)	90.0	90	0.0
γ (°)	120.02	120.0	0.0
*V* (Å^3^)	695.50	681.14	2.1
