# Supplementary material for: Role of lone-pair electron localization in temperature-induced phase transitions in mimetite
Source: Acta Crystallogr B Struct Sci Cryst Eng Mater. 2022 Jul 9;78(Pt 4):618–26. doi: 10.1107/S2052520622006254 (PMC9370212; doi:10.1107/S2052520622006254)
Supplement: Supplementary file 2 [file b-78-00618-sup2.pdf]

STRUCTURAL SCIENCE
CRYSTAL ENGINEERING
MATERIALS

Volume 78 (2022)

Supporting information for article:

Role of lone-pair electron localization in temperature-induced phase transitions in mimetite]

Georgia Cametti, Mariko Nagashima and Sergey V. Churakov

Table S1 Experimental details of mimetite at 173, 273, and 353 K.

	Mimetite-M (173 K)	Mimetite-M (273 K)	Mimetite-M (353 K)
**Crystal data**			
Chemical formula	$As_4Cl_{1.33}O_{16}Pb_{6.67}$	$As_3ClO_{12}Pb_5$	$As_4Cl_{1.33}O_{16}Pb_{6.67}$
Crystal system, space group	Monoclinic, $P112_1/b$	Monoclinic, $P112_1/b$	Monoclinic, $P112_1/b$
Temperature (K)	173	273	353
a, *b*, *c* (Å)	10.2378 (3), 20.4573 (7), 7.4457 (2)	10.2398 (4), 20.4774 (9), 7.4554 (3)	10.2544 (4), 20.5052 (7), 7.4568 (2)
α, β, γ (°)	90, 90, 120.039 (5)	90, 90, 119.981 (6)	90, 90, 119.971 (5)
V (Å^3)	1349.96 (9)	1354.10 (12)	1358.26 (10)
Z	3	4	3
Radiation type	Ag $K\alpha$, λ = 0.56087 Å	Ag $K\alpha$, λ = 0.56087 Å	Ag $K\alpha$, λ = 0.56087 Å
μ (mm^{-1})	37.69	37.57	37.71
Crystal size (mm)	0.10 × 0.06 × 0.04	0.10 × 0.06 × 0.04	0.10 × 0.06 × 0.04
**Data collection**			
Diffractometer	XtaLAB Synergy, Dualflex, Hypix	XtaLAB Synergy, Dualflex, Hypix	XtaLAB Synergy, Dualflex, Hypix
No. of measured, independent and observed [$I > 2\sigma(I)$] reflections	23244, 5558, 4813	27265, 5414, 4531	24325, 5534, 4410
R_{int}	0.046	0.047	0.038
$(\sin \theta/\lambda)_{max}$ (Å^{-1})	0.794	0.794	0.794
**Refinement**			
$R[F^2 > 2\sigma(F^2)]$, $wR(F^2)$, S	0.039, 0.086, 1.14	0.032, 0.071, 1.05	0.029, 0.069, 1.08
No. of reflections	5558	5414	5534
No. of parameters	192	190	190
	$w = 1/[\sigma^2(F_o^2) + (0.0142P)^2 + 76.360P]$ where $P = (F_o^2 + 2F_c^2)/3$	$w = 1/[\sigma^2(F_o^2) + (0.0218P)^2 + 19.508P]$ where $P = (F_o^2 + 2F_c^2)/3$	$w = 1/[\sigma^2(F_o^2) + (0.0266P)^2 + 2.9140P]$ where $P = (F_o^2 + 2F_c^2)/3$
$\Delta\rho_{max}$, $\Delta\rho_{min}$ (e Å^{-3})	4.44, −5.28	2.96, −4.32	4.22, -3.78

Table S2. Selected geometric parameters (Å) of mimetite-2M at 123 K.

Pb11—O19	2.46 (2)	Pb12—O112	2.48 (3)	Pb13—O18	2.40 (2)
Pb11—O17	2.48 (3)	Pb12—O110	2.53 (3)	Pb13—O19	2.52 (3)
Pb11—O18	2.54 (3)	Pb12—O111	2.57 (3)	Pb13—O27^{iii}	2.56 (2)
Pb11—O32	2.62 (2)	Pb12—O212	2.60 (2)	Pb13—O17	2.62 (3)
Pb11—O29^{i}	2.66 (2)	Pb12—O316^{i}	2.62 (3)	Pb13—O28^{iv}	2.65 (2)
Pb11—O320^{i}	2.72 (2)	Pb12—O211^{ii}	2.81 (2)	Pb13—O317^{iii}	2.72 (3)
Pb11—O28	3.00 (2)	Pb12—O210^{i}	2.82 (2)	Pb13—O29^{iii}	2.87 (3)
Pb11—O27^{i}	3.17 (2)	Pb12—O318^{ii}	3.10 (3)	Pb13—O321^{iii}	3.11 (2)
Pb11—O313^{i}	3.22 (3)			Pb13—O31^{iv}	3.18 (3)
Pb14—O110	2.42 (3)	Pb15—O12	2.38 (2)	Pb16—O14	2.44 (3)
Pb14—O111	2.50 (3)	Pb15—O11	2.56 (2)	Pb16—O16	2.47 (3)
Pb14—O312^{iv}	2.57 (2)	Pb15—O13^{iii}	2.57 (3)	Pb16—O33	2.53 (2)
Pb14—O112	2.62 (2)	Pb15—O310^{vi}	2.67 (3)	Pb16—O24^{viii}	2.56 (3)
Pb14—O322^{v}	2.76 (2)	Pb15—O323^{iii}	2.71 (3)	Pb16—O15	2.62 (3)
Pb14—O211^{v}	2.79 (2)	Pb15—O22^{vii}	2.72 (3)	Pb16—O26	2.71 (2)
Pb14—O210^{iii}	2.81 (2)	Pb15—O21^{iii}	2.79 (2)	Pb16—O25	2.91 (3)
Pb14—O212^{iv}	2.96 (2)	Pb15—O23^{vi}	3.11 (2)	Pb16—O34	3.28 (3)
Pb14—O38^{iii}	3.12 (3)	Pb15—O315^{vii}	3.12 (2)	Pb16—O39^{viii}	3.33 (3)
**Pb17**—O15^{iv}	2.46 (2)	Pb18—O11	2.51 (3)		
Pb17—O14^{iv}	2.48 (2)	Pb18—O13^{iii}	2.53 (3)		
Pb17—O16^{iv}	2.49 (3)	Pb18—O23^{ix}	2.57 (3)		
Pb17—O35	2.60 (2)	Pb18—O12	2.63 (3)		
Pb17—O37^{viii}	2.66 (3)	Pb18—O324^{x}	2.67 (2)		
Pb17—O25	2.71 (3)	Pb18—O21^{i}	2.75 (2)		
Pb17—O26	2.99 (2)	Pb18—O22^{x}	2.82 (3)		
Pb17—O24^{viii}	3.03 (3)	Pb18—O319^{i}	3.15 (3)		
Pb17—O314	3.35 (2)	Pb18—O311^{ix}	3.40 (3)		
Pb21—O23^{ix}	2.33 (2)	Pb22—O212	2.38 (2)	Pb23—O211	2.37 (2)
Pb21—O37^{xi}	2.50 (3)	Pb22—O35^{xi}	2.40 (3)	Pb23—O36^{xiii}	2.45 (2)
Pb21—O315^{x}	2.63 (3)	Pb22—O38^{i}	2.42 (3)	Pb23—O315^{xiii}	2.55 (3)

Pb21—O39	2.70 (3)	Pb22—O34	2.95 (3)	Pb23—O324^{xiv}	2.68 (3)
Pb21—O324^{x}	2.82 (2)	Pb22—O16	3.10 (2)	Pb23—O11^{i}	2.95 (3)
Pb21—O15^{ix}	3.05 (3)	Pb22—Cl2^{i}	3.120 (9)	Pb23—O312^{xiii}	3.07 (2)
Pb21—Cl4^{xii}	3.100 (8)	Pb22—O316^{i}	3.18 (2)	Pb23—Cl4^{xv}	3.130 (9)
Pb21—Cl4^{ii}	3.232 (8)	Pb22—Cl2	3.218 (9)	Pb23—Cl4^{viii}	3.187 (9)
Pb24—O27	2.35 (2)	Pb25—O24	2.41 (2)	Pb26—O28	2.32 (2)
Pb24—O323^{iii}	2.43 (3)	Pb25—O314^{ix}	2.41 (3)	Pb26—O33	2.55 (3)
Pb24—O313	2.44 (3)	Pb25—O310^{vi}	2.43 (3)	Pb26—O321^{i}	2.59 (2)
Pb24—O319^{i}	2.85 (3)	Pb25—O311^{ix}	2.88 (3)	Pb26—O314^{xi}	2.73 (3)
Pb24—O317	2.86 (2)	Pb25—O12	2.95 (3)	Pb26—O320^{i}	2.78 (2)
Pb24—O13^{iii}	2.97 (2)	Pb25—Cl1^{iii}	3.062 (8)	Pb26—O14	3.08 (3)
Pb24—Cl3	3.175 (8)	Pb25—O33^{ix}	3.11 (2)	Pb26—Cl1^{xvi}	3.141 (8)
Pb24—Cl3^{vii}	3.177 (7)	Pb25—Cl1^{ix}	3.258 (8)	Pb26—Cl1	3.156 (8)
Pb27—O26	2.35 (2)	Pb28—O34	2.37 (3)	Pb29—O316^{i}	2.34 (3)
Pb27—O39^{viii}	2.39 (3)	Pb28—O32	2.41 (3)	Pb29—O31^{iii}	2.39 (3)
Pb27—O36	2.49 (3)	Pb28—O25	2.42 (2)	Pb29—O29	2.42 (2)
Pb27—O312^{iv}	2.74 (3)	Pb28—O31^{iv}	2.86 (3)	Pb29—O38^{iii}	2.95 (3)
Pb27—O110	3.11 (2)	Pb28—O19	2.96 (2)	Pb29—O111	3.05 (3)
Pb27—Cl4^{iii}	3.118 (9)	Pb28—Cl2	3.040 (9)	Pb29—Cl2^{iii}	3.087 (8)
Pb27—Cl4^{xvii}	3.154 (9)	Pb28—Cl2^{iii}	3.229 (9)	Pb29—O32^{iii}	3.19 (3)
Pb27—O37^{viii}	3.24 (2)	Pb28—O35	3.33 (3)	Pb29—Cl2	3.190 (8)
P210—O22^{xiii}	2.29 (3)	P211—O210	2.32 (2)	P212—O21	2.35 (2)
P210—O322^{iv}	2.38 (3)	P211—O318^{xviii}	2.45 (3)	P212—O311^{xix}	2.36 (2)
P210—O319	2.46 (3)	P211—O317^{iii}	2.54 (2)	P212—O321^{iii}	2.42 (3)
P210—O318	2.90 (3)	P211—O313^{i}	2.75 (3)	P212—O320^{i}	2.87 (3)
P210—O112^{xiii}	3.08 (3)	P211—O18	3.08 (3)	P212—O17	2.94 (2)
P210—Cl3^{xiii}	3.134 (7)	P211—Cl3^{iii}	3.088 (7)	P212—Cl1	3.065 (9)
P210—Cl3^{iii}	3.162 (7)	P211—Cl3^{xiii}	3.168 (7)	P212—Cl1^{xix}	3.189 (8)
P210—O323	3.19 (3)	P211—O322^{xviii}	3.22 (3)	P212—O310^{xix}	3.29 (2)
				P212—O314	3.47 (2)

As1—O38	1.68 (3)	As11—O112	1.64 (2)	As2—O323	1.66 (3)
As1—O210	1.69 (3)	As11—O317	1.65 (2)	As2—O319	1.68 (3)
As1—O316	1.71 (2)	As11—O29	1.69 (3)	As2—O18	1.72 (2)
As1—O19	1.76 (2)	As11—O313	1.69 (3)	As2—O21	1.74 (2)
As21—O320	1.67 (3)	As3—O111	1.64 (2)	As31—O15	1.67 (2)
As21—O27	1.68 (3)	As3—O35	1.68 (3)	As31—O312	1.69 (3)
As21—O321	1.69 (3)	As3—O26	1.69 (2)	As31—O36	1.70 (3)
As21—O12	1.75 (2)	As3—O34	1.70 (3)	As31—O212	1.71 (3)
As4—O324	1.68 (3)	As41—O318	1.61 (3)	As5—O25	1.63 (2)
As4—O110	1.69 (3)	As41—O211	1.62 (2)	As5—O314	1.66 (3)
As4—O315	1.72 (3)	As41—O13	1.67 (3)	As5—O17	1.69 (3)
As4—O22	1.77 (2)	As41—O322	1.74 (3)	As5—O33	1.71 (3)
As51—O32	1.66 (3)	As6—O310	1.67 (3)	As61—O11	1.66 (3)
As51—O16	1.70 (3)	As6—O311	1.70 (3)	As61—O24	1.69 (3)
As51—O31	1.74 (3)	As6—O23	1.70 (3)	As61—O39	1.69 (3)
As51—O28	1.78 (2)	As6—O14	1.71 (3)	As61—O37	1.74 (3)

Symmetry codes: : (i) −*x*+1, *y*+1/2, −*z*+1; (ii) *x*, *y*, *z*+1; (iii) −*x*+1, *y*−1/2, −*z*+1; (iv) *x*, *y*−1, *z*; (v) *x*, *y*−1, *z*+1; (vi) −*x*+1, *y*−1/2, −*z*+2; (vii) *x*−1, *y*−1, *z*; (viii) *x*+1, *y*, *z*; (ix) −*x*+1, *y*+1/2, −*z*+2; (x) *x*−1, *y*, *z*; (xi) *x*, *y*+1, *z*; (xii) −*x*, *y*+1/2, −*z*+1; (xiii) *x*, *y*, *z*−1; (xiv) *x*, *y*+1, *z*−1; (xv) −*x*+1, *y*+1/2, −*z*; (xvi) −*x*+2, *y*+1/2, −*z*+1; (xvii) *x*+1, *y*, *z*+1; (xviii) −*x*+1, *y*−1/2, −*z*; (xix) −*x*+2, *y*−1/2, −*z*+1; (xx) *x*+1, *y*+1, *z*; (xxi) −*x*, *y*−1/2, −*z*+1; (xxii) *x*−1, *y*, *z*−1.”
